# Ixchiq (VLA1553): The first FDA-approved vaccine to prevent disease caused by Chikungunya virus infection

**DOI:** 10.1080/21505594.2023.2301573

**Published:** 2024-01-13

**Authors:** Hinh Ly

**Affiliations:** Department of Veterinary & Biomedical Sciences, College of Veterinary Medicine, University of Minnesota, Twin, MN, USA

**Keywords:** Vaccine, chikungunya virus, CHIKV, Alphavirus, arbovirus, Ixchiq (VLA1553)

Chikungunya, a disease caused by the arbovirus Chikungunya virus (CHIKV), a member of the mosquito-borne Alphavirus genus in the *Togaviridae* family, poses a substantial health burden in Africa, Southeast Asia, and some parts of the America and Europe [1]. Although the virus was rarely found in the USA, recent studies have reported a couple dozen cases in US travellers between 2006 and 2013 and cases of local transmissions reported in Florida, Texas, Puerto Rico and the US Virgin Islands in late 2014, raising public health concerns for US travellers and those living in warmer locales [2]. The World Health Organization (WHO) considers chikungunya an emerging threat to global health that is responsible for at least 5 million cases in the past 15 years [3], although deaths and severe illness due to CHIKV infections are relatively rare.

While 3% to 28% of CHIKV-infected individuals are asymptomatic, Chikungunya patients can experience fever, headache, and a skin rash, with about 20–30% of the patients developing severe and long-lasting joint pain and muscle pain (polyarthralgia and myalgia), and hence, the name Chikungunya which means “bending over in pain” in the Makonde dialect of Africa. The acute phase of the disease can last for about a week, which can be followed by a chronic phase that is characterized by severe and persistent joint pain with occasional ophthalmic, neurological, and cardiac complications [4]. Chikungunya has no specific treatment, except for supportive cares, and can be debilitating and even deadly for newborns. Some neonatal cases can be associated with encephalitis. Neonates as well as the elderly bear a more substantial disease burden, with mortality rates being disproportionately higher in individuals aged 65 and above and underscoring the age-dependent disease severity.

CHIKV, first discovered in 1952 in the United Republic of Tanzania (for an epidemiological review, see [1]) as an RNA virus with a positive single-strand genome of about 11.6 kb, has received global attention due to its inclusion in the Coalition for Epidemic Preparedness Innovations (CEPI) list of priority pathogens for vaccine development [5]. The virus exhibits dual transmission cycles, urban and sylvatic, with the former involving human-to-mosquito-to-human transmission [6]. Therefore, the main preventative strategies were to avoid mosquito bites and to eliminate mosquito-breeding grounds, since no specific prophylactic or therapeutic vaccines and drugs were available. Numerous vaccines against chikungunya are in Phase II and III clinical-trial developments [7]. Among these are the Valneva’s single-dose live-attenuated Ixchiq (VLA1553) vaccine that has recently obtained Food and Drug Administration (FDA) approval for use [8].

Ixchiq (VLA1553) is a live, attenuated vaccine that contains a weakened version of the CHIKV that may still cause symptoms that are similar to natural CHIKV infection. The presence of the weakened version of the virus that makes up the Ixchiq (VLA1553) vaccine could be detected in the blood of some individuals in the first few weeks after they were vaccinated, akin to what were seen in the early days of CHIKV (natural) infection. Because of this, Ixchiq (VLA1553) vaccine recipients are advised as to the current lack of information about whether the vaccine virus can be transmitted from a pregnant person to their newborn (vertical transmission) and whether it can cause foetal harms (congenital infection). The FDA recommends healthcare providers and their patients to weigh the risk-benefit factors as well as the gestational age and risks of the Ixchiq (VLA1553) vaccination to the foetus or neonate from the disease. The FDA also requires the vaccine maker Valneva to do a postmarket study to assess the potential long-term risks of the vaccine.

The Ixchiq (VLA1553) vaccine was administered as a single intramuscular injection and had undergone evaluation for safety and immunogenicity in a double-blind, randomized, placebo-controlled, phase 3 clinical trial. This trial was conducted at 43 sites in the USA and involved 4,115 healthy participants, who were randomly assigned (3:1) to receive either the Ixchiq (VLA1553) vaccine or placebo [9]. The results assessing the immunogenicity of the vaccine in a per-protocol population of 362 participants (266 in the vaccinated group and 96 in the placebo group) showed that following a single dose of vaccination, the Ixchiq (VLA1553) could elicit CHIKV-specific neutralizing antibody levels in 98.9% of the vaccinated individuals. The Ixchiq (VLA1553) vaccine demonstrated a generally safe profile with uniform tolerability across age groups tested. As with natural CHIKV infections, the most common side effects reported in those vaccine studies submitted to the FDA for vaccine’s approval included fatigue (tiredness), headache, muscle and joint pain, fever, and tenderness at the injection site. Less than 2% of people who received the vaccine had severe chikungunya-like adverse reactions that required medical intervention. Serious adverse events were reported in 46 (1.5%) of 3,082 participants who received the vaccine and in eight (0.8%) of 1,033 participants in the placebo arm. Notably, only two of the nearly 3,500 people in the trials required hospitalization due to some adverse reactions, such as myalgia and syndrome of inappropriate antidiuretic hormone secretion. Some also had a chikungunya-like adverse reaction that lasted at least 30 days. Except for a limited number of reported adverse events due to vaccination, the Ixchiq (VLA1553) vaccine was overall deemed to be safe and efficacious against chikungunya.

Recent outbreaks of CHIKV infections, such as the 2004–2007 chikungunya epidemic that affected millions of people living in the Indian Ocean, Europe, and the Americas, and caused significant morbidity and mortality [7], served as a wake-up call for continuing global surveillance and research into the disease transmission, pathology, pathogenesis, and treatment modalities. Diagnostic (serological) methods, such as enzyme-linked immunosorbent assays (ELISA) that can detect the presence of IgM and IgG anti-CHIKV antibodies (especially during the initial days of the infection), and vaccine [e.g. the Ixchiq (VLA1553) vaccine] and therapeutic developments along with traditional means of disease prevention, such as mosquito controls, need to be considered as comprehensive strategies to curtail the global public health threat of CHIKV infections.

## Data Availability

No primary data is included in this article.
